# Determination of Edema in Porcine Coronary Arteries by T2 Weighted Cardiovascular Magnetic Resonance

**DOI:** 10.1186/1532-429X-13-52

**Published:** 2011-09-21

**Authors:** Steen Fjord Pedersen, Samuel A Thrysøe, William P Paaske, Troels Thim, Erling Falk, Steffen Ringgaard, Won Yong Kim

**Affiliations:** 1 Dept. of Cardiothoracic and Vascular Surgery T, Aarhus University Hospital Skejby, Brendstrupsgaardsvej 100, DK-8200 Aarhus N, Denmark; 2 Dept. of Cardiology, Aarhus University Hospital Skejby, Brendstrupsgaardsvej 100, DK-8200 Aarhus N, Denmark; 3 MR-center, Aarhus University Hospital Skejby, Brendstrupsgaardsvej 100, DK-8200 Aarhus N, Denmark

## Abstract

**Background:**

Inflammation plays a pivotal role in all stages of atherosclerosis. Since edema is known to be an integral part of inflammation, a noninvasive technique that can identify edema in the coronary artery wall may provide unique information regarding plaque activity. In this study, we aimed to determine whether edema induced in porcine coronary arteries by balloon injury could be reliably detected by cardiovascular magnetic resonance (CMR) using a water sensitive T2-weighted short tau inversion recovery sequence (T2-STIR). We also aimed to compare these results to those of conventional T2-weighted (T2W) imaging.

**Methods:**

Edema was induced in the proximal left anterior descending (LAD) coronary artery wall in seven pigs by balloon injury. At baseline, and 1-10 days (average four) post injury, the proximal LAD was assessed by water sensitive T2-STIR and conventional T2W sequences in cross-sectional planes. CMR images were matched to histopathology, validated against Evans blue as a marker of increased vessel wall permeability, and correlated with the arterial amount of fibrinogen used as an edema surrogate marker.

**Results:**

Post injury, the T2-STIR images of the injured LAD vessel wall showed a significant 72%, relative signal intensity (SI) increase compared with baseline (p = 0.028). Using a threshold value of SI 7 SD above the average SI of the myocardium, T2-STIR detected edema in the vessel wall (i.e. enhancement) with a sensitivity of 100 and a specificity of 71. Twelve out of the 14 (86%) T2-STIR images displaying coronary artery wall enhancement also showed Evans blue uptake in the corresponding histology. The relative signal intensity showed a linear correlation with the amount of fibrinogen detected on the corresponding histopathology (ρ = 0.750, p = 0.05). The conventional T2W images did not show significant changes in SI post injury.

**Conclusion:**

T2-STIR CMR enabled detection of coronary artery wall edema and could therefore be a non-invasive diagnostic tool for evaluation of inflammatory coronary artery wall activity.

## Background

Atherosclerosis is the main cause of coronary artery disease (CAD), which remains the leading cause of premature death and morbidity in the western world [1]. Over the past decades, it has become evident that inflammation plays a key role not only for the initiation and progression of atherosclerosis, but also for atherosclerotic plaque destabilization and rupture [2-5]. A non-invasive imaging technique that can detect the presence of inflammation within the coronary artery wall (CAW) may therefore offer many enticing prospects, including early coronary atherosclerosis detection, identification of patients at risk of plaque rupture, assessment of treatment response, and further insights into the biology of coronary atherosclerosis.

The challenge in non-invasive CAW inflammation imaging is rooted in the small caliber of the CAW, in cardiac and respiratory motion, and in the lack of clinically approved imaging markers suitable for detecting inflammation. However, cardiovascular magnetic resonance (CMR) allows high-resolution non-invasive CAW imaging when combined with cardiac and respiratory motion compensation techniques [6-8]. Previous studies have documented the ability of this techniques to detect inflammation within larger artery walls, e.g. in the carotid artery, using gadolinium contrast agents and iron oxide particles [9-12]. However, none of these contrast agents have been demonstrated in CAW imaging and none of these contrast agents seem close to clinical approval. Therefore there are currently no clinically suitable contrast agents available for use in CAW inflammation detection.

To overcome these problems and bring non-invasive CAW inflammation detection closer to clinical practice, a non-contrast-dependent CMR approach is preferable. We hypothesize that edema may be a useful marker for CAW inflammation since edema is an integral component of inflammation [13] that has previously been shown to be traceable with CMR in aortitis and arteritis using a short tau inversion recovery sequence (T2-STIR) to suppress the signal from perivascular fat and enhance the signal from water [14-16]. Recently, we demonstrated by CMR T2-STIR that local coronary vessel wall edema was present in the culprit lesion of a patient with acute coronary syndrome [17].

In this study, we aimed to determine whether balloon over-extension causing edema and inflammation in porcine coronary arteries [18] could be reliably detected using CMR with water sensitive T2-STIR. We also aimed to compare these results to those of a standard T2W sequence used for CMR assessment of carotid atherosclerosis [19].

## Methods

### Animal Model

**Figure 1:**
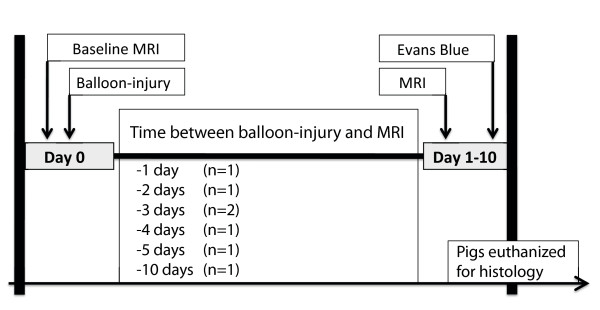
**Study design, showing the time-line of the CMR experiment of imaging coronary vessel wall edema**. Balloon overstretch injury was induced in the LAD of 40 kg Danish Land Race pigs (n = 7). Cross sectional views of the LAD were acquired by CMR before and post balloon injury. The animals were euthanized to correlate CMR observations with histopathological findings. The number of animals assessed by CMR at various time points after the balloon injury is indicated in parenthesis.

Seven female Danish Land Race pigs weighing 40 kg were used for the experiments (Figure 1). All the pigs were treated in full accordance with the Danish law on animal experiments.

The pigs were pre-sedated with an intramuscular injection of stressnill (1 ml/kg), and midazolam (1 ml/kg). After induction of anesthesia with intravenous propofol (5 mg/kg) and endotracheal intubation, anesthesia was maintained with continuous intravenous infusion of propofol (8-10 mg/kg/hr) and fentanyl (2 mg/kg/hr). The pigs were mechanically ventilated with a tidal volume of 450 ml (respiratory rate 12/min).

The right common femoral artery was exposed by a surgical cut down, and a 9 F introducer sheath was inserted into the artery followed by a bolus injection of 100 IU/kg heparin administrated through the sheath. For the balloon overstretch injury, a 4.5 mm over-the-wire angioplasty balloon was placed in the LAD just distal to the origin of the circumflex artery. The injury was induced by two inflations, each at 12 atmospheres, and each lasting 30 seconds and with 60 seconds between the inflations. The inflation ensured a balloon to artery ratio of 1.6 to 1.8.

Acetylsalicylic acid (100 mg/d) was given orally after the procedure and continued until euthanasia was induced by phenobarbital.

### Cardiovascular Magnetic Resonance

CMR was performed at baseline and one to 10 days (average 4) following coronary balloon injury depending on scanner availability and access to the animal laboratory (Figure 1). CMR was performed on a 1.5T MR system (Intera, Philips Medical Systems, Best, The Netherlands) using a five-element cardiac synergy coil with the same sedation and respiratory protocol as described above in the animal model section. After a survey scan to localize the heart and diaphragm, a multi-heart phase steady-state free precession (SSFP) cine scan (repetition time (TR) 2.6 ms; echo time (TE) 1.3 ms; flip angle 60°; 50 heart phases; SENSE factor 2) was obtained in the 4-chamber view to assess the interval of minimal LAD motion and determine the optimal diastolic trigger delay for the subsequent coronary scans [20].

A non-contrast-enhanced, bright-blood coronary angiography was performed with a previously described navigator-gated, free-breathing and cardiac-triggered T2 prepared 3D SSFP sequence allowing for visualization of the LAD and circumflex coronary artery lumen (TR 4.9 ms; TE 2.5 ms; flip angle 90°) [21].

Subsequently, T2-weighted CMR was performed in cross-sections of the LAD just distal to the origin of the circumflex artery. The parameters for the navigator-gated, free-breathing, ECG-triggered, dark-blood, T2-weighted short-tau inversion-recovery (STIR), fast-spin echo sequence were as follows: TR 2 RR intervals; TE 100 ms, echo train length of 20 yielding a total acquisition time of 180 ms, and 6 averages, TI 174 ms for the STIR prepulse, the prepulse and imaging sequence was implemented within the same RR interval. The parameters for the ECG-triggered, dark-blood, standard T2-weighted sequence were as follows: TR 2 RR intervals; TE 40 ms, echo train length of 5, and 2 averages. The balloon-injured segment of the LAD was encompassed by 2 contiguous slices (0.68 × 0.68 × 5 mm^3 ^voxels).

### Histopathology

After intravenous injection, virtually all Evans blue dye binds to albumin and only enters the vessel wall in significant amounts in the presence of a defect endothelial barrier [22-24]. In this study, the dye was used to ease the localization of the balloon-injured LAD segment and to macroscopically verify that the balloon injury had caused increased vessel wall permeability. The solution was prepared by dissolving 2 g of Evans blue (Serva, Heidelberg, Germany) in 50 ml of isotonic saline. The Evans blue dye was administered over 1 hour through an ear vein cannula and allowed to circulate in the bloodstream for 1 hour, after which the animals were killed and the hearts were harvested. The proximal LAD was removed from the heart and cut into 5 mm consecutive cross sectional slices and subsequently photographed with a digital camera (Nikon, Tokyo, Japan) for the purpose of registering any uptake of Evans blue dye. Surrounding epicardial fat and myocardium were included in the section for arterial support during fixation and to improve matching with the CMR images through the use of anatomic markers. The LAD slices covering the balloon-injured area (2 per pig) were fixated in formaldehyde and embedded in paraffin. Fibrinogen was used as a surrogate marker for edema [13,25] and identified by staining 6 μm deparaffinized sections with rabbit anti-human fibrinogen antibody (A0080; Dako, Glostrup, Denmark; 1:1000 dilution) for 12 hours. Unspecific binding was blocked with normal goat serum (X0907; Dako, Glostrup, Denmark; 1:5 dilution) for 20 minutes, followed by staining for 30 minutes with polyclonal goat anti-rabbit secondary antibody (E0432; Dako, Glostrup, Denmark; 1:300 dilution).

### Data analysis

#### CMR

The arterial lumen and outer vessel wall boundary were manually defined on the T2-STIR images using Matlab R2009a (Mathworks Inc, Natick, MA, USA). A Region of Interest (ROI) was placed in a homogenous region of the myocardium and the relative mean signal intensity (SI) was calculated as: SI_vessel wall_/SI_myocardium_. To establish the optimal SI threshold level criteria that would allow us to differentiate between injured and non-injured LAD segments, the artery wall enhancement area was assessed from three to eight SD above the mean SI of the normal myocardium.

The relative mean SI of the coronary artery wall was measured at baseline and post injury, and the presence or absence of enhancement was determined for each of the images by two observers (SFP, ST).

#### Histopathology

Two observer (SFP, ST) reviewed all the digital photos of the freshly cut LAD slices and categorized each segment as being either positive or negative for Evans blue uptake based on the presence or absence of a distinct blue color in the vessel wall and perivascular area. The histological sections stained for fibrinogen were reviewed by two observers (SFP, WYK).

#### Fibrinogen staining (edema)

To determine the edematous area of the vessel wall, the fibrinogen staining area (i.e., areas with a red color) and the vessel wall with surrounding adventitia were manually defined using ImageJ software (National Institutes of Health). The edematous area (in percentage of vessel wall area) was calculated as:

$$(\text{fibrinogen staining area }[{\text{mm}}^{\text{2}}]\text{/vessel wall area }[{\text{mm}}^{\text{2}}])\times \text{100}\%$$

#### CMR T2-STIR vs. histology

CMR images of the balloon-injured LAD were matched with the corresponding histological sections by using landmarks, such as the distance from the origin of the circumflex artery, and gross anatomical features, such as the shape and size of the coronary lumen and vessel wall. The relative SI on T2-STIR images was correlated to the area of fibrinogen detected in the corresponding histology. The occurrence of vessel wall enhancement detected on T2-STIR images was checked against the occurrence of Evans blue.

#### Statistical Analysis

Statistical differences in SI measured on water sensitive T2-STIR and standard T2W images at baseline and post injury were assessed using a using a Wilcoxon signed rank test. The correlation of relative SI and fibrinogen density was assessed by a Spearman ρ correlation test. Inter- and intraobserver reproducibility was assessed with a 1-way random, single-measure intraclass correlation coefficient (ICC) using SPSS software version 18.0 (SPSS Inc. Chicago, IL)

## Results

### CMR T2-STIR

All the CMR scans were successfully performed. Pre- and post-injury cross-sectional CMR images were obtained of the proximal LAD using the T2-STIR and T2W sequences, giving a total of 2 times 28 images (4 images pre- and post-injury per pig).

**Figure 2:**
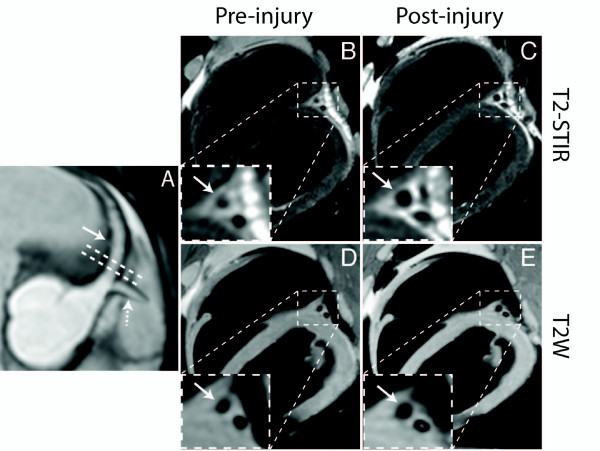
**In-plane CMR image (A) showing the LAD (arrow) and circumflex (dashed arrow) arteries**. The dashed lines show the location of the acquired fat-suppressed T2-STIR (B, C) and T2W (D, E) cross-sectional LAD imagesprior to balloon injury and three days post injury. Note the increased signal intensity of the LAD vessel wall post injury (arrow) by the T2-STIR images compared to the constant signal intensity of the T2W images.

**Figure 3:**
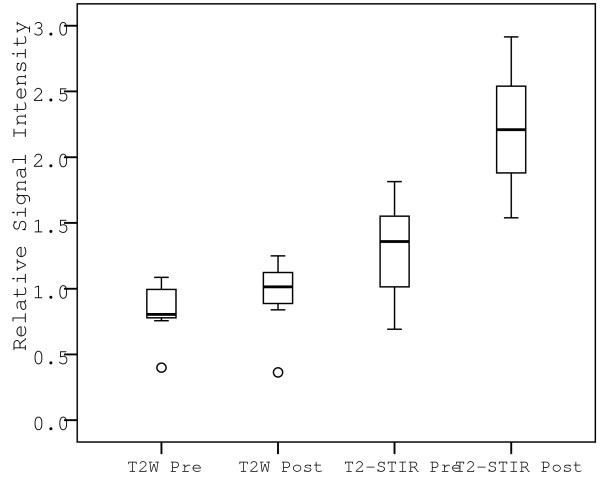
**The mean relative SI defined as: (*SI_vessel wall_/SI_myocardium_*) was detected pre and post injury in the proximal LAD of the six pigs using standard T2W and water sensitive T2-STIR sequences**. The bottom and top of the box shows the 25^th ^and 75^th ^percentile, respectively. The horizontal line inside the boxes represents the median and the end of the whiskers represents the minimum and maximum values and the circles represent outliers.

Post injury, T2-STIR of the CAW showed a 72% increase in relative SI compared to baseline (p < 0.028) while T2W imaging showed a non-significant 13% increase in relative SI (p = 0.178) (Figures 2 and 3).

According to a threshold level of 7 SD above the average signal intensity measured in the myocardium, the T2-STIR images showed enhancement in 3 out of 14 (21.4%) segments pre injury and in 14 out of 14 (100%) segments post injury resulting in a sensitivity of 100% and specificity of 71% (Table 1). The optimal threshold level for the T2W images was 2SD resulting in a modest sensitivity of 71% and specificity of 71%.

**Table 1:** Sensitivity and specificity for the detection of coronary vessel wall edema (i.e., artery wall enhancement) using water sensitive T2-STIR and standard T2W sequences at enhancement thresholds ranging from SI 1 to 8 SD above the SI of the myocardium.

	T2-STIR								T2W							
**Threshold **	**1**	**2**	**3**	**4**	**5**	**6**	**7**	**8**	**1**	**2**	**3**	**4**	**5**	**6**	**7**	**8**

Sensitivity	100	100	100	100	100	100	100	85	92	71	62	38	23	15	8	7

Specificity	29	29	29	36	57	64	71	71	46	71	84	84	1000	100	100	100

Accuracy	64	64	64	68	79	82	86	79	69	76	73	61	61	58	53	53

### Histopathology

Of the LAD segments that were exposed to balloon injury, 12 out of 14 (86%) showed uptake of Evans blue. For each pig, two histological sections were stained for fibrinogen, giving a total of 14 sections. Two of the histological sections were damaged, which left 12 sections for further analysis. Positive fibrinogen staining was observed in the vessel wall and surrounding adventitia in 11 of the 12 (92%) histological sections, with a mean fibrinogen area (in percentage of the vessel wall area) of 22%; CI95 = [14%-30%].

### CMR vs. Histology

**Figure 4:**
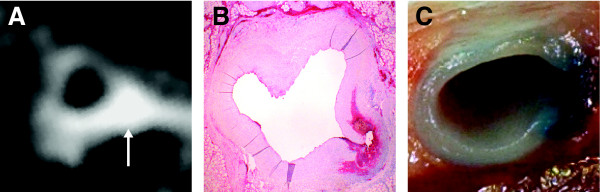
**Cross-sectional fat-suppressed T2-STIR image of the LAD post balloon injury showing enhancement (arrow) of the vessel wall (A)**. Photo of the corresponding segment showing extravasation of Evans blue (distinct blue color) into the arterial wall (B). Histological identification of fibrinogen (distinct red areas) at (×1.5) (C).

**Figure 5:**
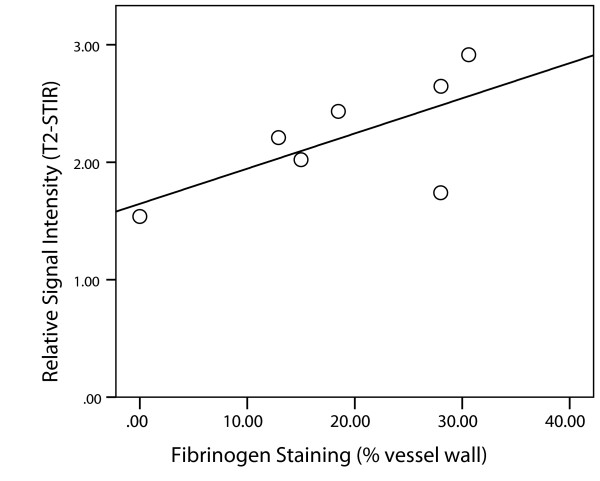
**Mean fibrinogen staining of the balloon-injured LAD segment versus the mean relative SI on the corresponding T2-STIR images**. For each of the seven pigs, all histologic sections stained for fibrinogen were averaged to generate a mean per- pig value for percentage fibrinogen staining. Likewise, the corresponding T2- STIR images were combined to generate a mean relative SI for the balloon-injured LAD segment. There was a non significant correlation between mean fibrinogen staining and mean relative SI (ρ = 0.750, p = 0.05).

Eleven of the fourteen (79%) segments that showed coronary artery wall enhancement (defined as SI that was seven SD above the average signal intensity measured in the myocardium) also showed uptake of Evans blue (Figure 4). The relative signal intensity showed a linear correlation to the area of fibrinogen detected on the corresponding histopathology (ρ = 0.75, p = 0.05) (Figure 5).

### Interobserver and Intraobserver Agreement

The reproducibility for the assessment of the average SI on cross sectional CMR images was, 0.97 (p < 0.001, CI_95 _= [0.92 - 0.99]) for interobserver agreement and 0.96 (p < 0.001, CI_95 _= [0.91- 0.98]) for intraobserver agreement. The reproducibility for the presence of fibrinogen (expressed as percentage of the total vessel wall area) was 0.99 (p = 0.0001), CI_95 _= [0.97- 0.998]) for interobserver agreement and 0.95 (p = 0.0001), CI_95 _= [0.960 - 0.996]) for intraobserver agreement.

## Discussion

The results of this study showed that a significant increase in signal intensity and thereby an enhancement of the porcine CAW could be detected by water sensitive T2-STIR imaging following balloon injury, whereas the T2W images showed no significant increase in signal intensity. Furthermore, we found the observed changes on T2-STIR images to correlate well with the presence and extension of the vessel wall edema detected by histology.

To the best of our knowledge, these results provide the first solid evidence that CMR T2-STIR, used with the motion compensation techniques as proposed, allows for non-invasive and in-vivo detection of CAW edema. Our recent studies in patients with acute ST-elevation myocardial infarction demonstrated the occurrence of coronary vessel wall edema within the culprit lesion [17]. Therefore, it appears that coronary vessel wall edema as a marker of inflammation may facilitate detection of thrombosis-prone coronary plaques. Furthermore, our unpublished observations of carotid atherosclerosis show edema in carotid plaques in asymptomatic patients suggesting that edema is present also during the build-up of atherosclerosis.

The observed ability of the T2-STIR technique to detect edema is consistent with previous studies that have found the same technique to be highly sensitive in detecting edema in arteritis [14-16]. T2-STIR sequence has also been successfully used to assess myocardial edema secondary to coronary artery occlusion [26]. One major strength of our study was that the CMR T2-STIR imaging results were validated directly against the occurrence of edema detected by histology. Histological detection of edema is difficult due to the lack of a histological method that directly can assess the presence of edema. In our study, vessel wall fibrinogen and albumin were used as surrogate markers for edema. The rationale behind this approach was that these proteins are normally restricted to the vessel lumen, but following vessel wall injury with increased vessel wall permeability they extravasate into the vessel wall and surrounding tissue together with the edema-forming fluid. We assessed the edema microscopically using immunohistochemistry to detect the occurrence of fibrinogen. We also checked macroscopically for Evans blue dye, which binds to albumin and therefore only enters the vessel wall where there is increased permeability and most likely edema. Both methods have been used previously in other studies to assess increased vessel wall permeability and edema in the vessel wall [25,27]. These methods are therefore considered feasible and, indeed, recommendable methods for validation of the presence of edema.

In this study, it took us 7-14 minutes (depending on the pig's heart rate) to scan the proximal LAD. It would therefore be time consuming to assess the whole coronary arterial tree using this technique. However, CAD is a diffuse process and the LAD is almost always to some extent involved if CAD is present. The proximal LAD should therefore be an adequate sample site for evaluating the presence and severity of CAD.

Another major advantage of using the CMR T2-STIR technique described in this study is that no contrast agents, special hardware, or special software are required. Clinical implementation of the proposed approach for identifying edema in the CAW should therefore pose no major problems. However, it requires further investigation to ascertain to which extent edema is a good marker for the inflammatory activity within atherosclerosis.

With this study we were able to prove the concept that edema can be detected non-invasively and *in vivo *by CMR T2-STIR.

### Limitations

No images were acquired in segments more distant to the injury, since the vessel dimensions become marginal for consistent visualization. However, a significant signal increase on T2-STIR images was observed following balloon injury compared to baseline scans.

Residual coronary motion may be present even with ECG triggering and the use of respiratory navigator to reduce respiratory motion. Thus, blurring of the coronary vessel wall due to coronary motion may occur. However, since both pre- and post-scans experience similar coronary motion during the experimental conditions, blurring is considered less of a problem. The reproducibility of the coronary edema scan in patients should be addressed in future studies.

Given the large disparity in slice thickness (4 μm for histology and 5 mm for CMR), matching CMR images to histology is challenging and associated with a certain level of inaccuracy, even with careful matching. To improve the accuracy of matching, we induced the balloon injury in the proximal LAD immediately distal to the origin of the circumflex artery. In this way, we had a landmark that made it possible to identify the balloon injury both on the CMR images and in the histopathology. In addition, we used Evans blue dye to further improve identification of the balloon-injured coronary segment. We therefore believe that we have duly catered for this problem.

## Conclusion

The T2-STIR sequence allowed for detection of edema in the balloon injured LAD with high sensitivity and specificity. Thus, this approach may identify edema caused by inflammation in the coronary arteries in patients suffering from CAD. Standard T2W imaging appeared unsuitable for edema detection in the CAW.

Further research in CAD patients will be required to establish the value of the T2-STIR approach for identifying edema/inflammation in atherosclerotic plaques.

## Competing interests

The authors declare that they have no competing interests.

## Authors' contributions

SFP carried out all the procedures related to the animal model performed all the CMR scans, analyzed the acquired CMR and histological data, contributed to the statistical analyses, obtained all illustrations, wrote the manuscript, and merged all feedback from the co-authors into the final manuscript. ST contributed to the analysis of the acquired CMR and histological data, the statistical analyses, the illustrations and drafting the manuscript and revised it critically for intellectual content. WP took part in formulating the study, revised the manuscript critically and contributed to important intellectual content of the manuscript. TT contributed to procedures related to the animal model, and drafting the manuscript and revised it critically for intellectual content. EF contributed to the study design, drafting the manuscript, and revised it critically, for intellectual content. SR contributed to the CMR sequence setup, revised the manuscript critically and contributed to important intellectual content of the manuscript. WYK contributed to the study design, the CMR sequence setup, drafting the manuscript and revised it critically for intellectual content. All authors have read and approved the final manuscript.
